# Evaluation of Antitumor and Antimicrobial Photobiological Activity of Nanocarrier Containing Photosensitizer and Magnetic Nanoparticle

**DOI:** 10.3390/cimb48030324

**Published:** 2026-03-19

**Authors:** Raphaela Aparecida Schuenck Rodrigues, Sandro Pinheiro da Costa, Veronica da Silva Cardoso, Alane Beatriz Vermelho, Ralph Santos-Oliveira, Franklin Chimaobi Kenechukwu, Eduardo Ricci-Junior

**Affiliations:** 1 Laboratório de Desenvolvimento Galênico (LADEG), Farmácia Universitária, Universidade Federal do Rio de Janeiro (UFRJ), Rio de Janeiro 21941-901, Brazil; raphapharma@gmail.com (R.A.S.R.); sandropinheiropharma@gmail.com (S.P.d.C.); 2 Laboratório de Biocatálise, Bioprodutos e Bioenergia (BIOINOVAR), Universidade Federal do Rio de Janeiro (UFRJ), Rio de Janeiro 21941-901, Brazil; verocardoso@micro.ufrj.br (V.d.S.C.); abvermelho@micro.ufrj.br (A.B.V.); 3 Laboratory of Nanoradiopharmacy and Synthesis of Novel Radiopharmaceuticals, Nuclear Engineering Institute, Rio de Janeiro 21941-598, Brazil; presidenciaradiofarmacia@gmail.com; 4 Laboratory of Nanoradiopharmacy and Radiopharmaceuticals, Zona Oeste State University, Rio de Janeiro 20550-013, Brazil; 5 Drug Delivery and Nanomedicines Research Group, Department of Pharmaceutics, Faculty of Pharmaceutical Sciences, University of Nigeria, Enugu 410105, Nigeria; frankline.kenechukwu@unn.edu.ng

**Keywords:** nanoparticles, photosensitizer, photobiological activity, photodynamic therapy, antitumor activity, antimicrobial activity

## Abstract

Nanotechnology combined with photodynamic therapy (PDT) has been explored to enhance antitumor and antimicrobial photobiological activity. Aluminum phthalocyanine chloride (Al-Pc-Cl), with or without magnetic nanoparticles (MagNPs), was incorporated into polymeric nanoparticles (PNPs) to improve the PDT for treating tumors and infectious diseases. Three batches of the nanoparticles (MagNPs, PNPs-PS and PNPs-PS-MagNPs) were developed and characterized in terms of size, PdI, morphology by TEM, release study, and antitumor (against A549 cells) and antimicrobial (against MRSA and *C. albicans*) photobiological activity. The developed nanoparticles were nanometric in size, with MagNPs, PNPs-PS, and PNPs-PS-MagNPs showing 33.6, 186.9, and 333.5 nm, respectively, maintained the magnetic properties (for MagNPs and PNPs-PS-MagNPs), and provided slow and sustained release of the photosensitizer. PNPs-PS and PNPs-PS-MagNPs showed excellent antitumor photobiological activity with cell viabilities of 42 and 34%, respectively, and were not cytotoxic in the dark, with cell viabilities above 70%. PNPs-PS showed strong antibacterial activity against MRSA with an IC_50_ of 8.26 μg/mL, which was lower to free Al-Pc-Cl with an IC_50_ of 14.22 μg/mL after I radiation. The results of the antifungal photobiological activity against *C. albicans* were excellent, with IC_50_ values of 3.75 and 3.5 μg/mL for PNPs-PS and PNPs-PS-MagNPs, respectively, values which were significantly lower with *p* < 0.05 than free PS (IC_50_ > 30 μg/mL) after irradiation with light and fluconazole (IC_50_ > 30 μg/mL), the reference antifungal agent. PNPs-PS showed promising results regarding antitumor, antibacterial, and antifungal photobiological activity. However, PNPs-PS-MagNPs showed weak results for antibacterial photobiological activity against MRSA but with promising results for tumor cells and *C. albicans*.

## 1. Introduction

Photodynamic therapy (PDT) has been used in the treatment of various diseases, including cancer and infectious diseases caused by bacteria, fungi and parasites. The mechanism involved in this therapy is the activation of a photosensitizer by visible light in the presence of molecular oxygen, resulting in a cytotoxic effect. The selectivity of this treatment is related to the low cytotoxicity of the photosensitizer in the absence of light and its strong photobiological activity in the presence of visible light, so that only cells simultaneously exposed to both the photosensitizer and light are eliminated [1,2,3,4,5]. PDT has been approved for the treatment of cancers of the esophagus, lung, head and neck, skin, and bladder [3,5,6].

The three main components of PDT are the photosensitizer (PS), visible light, and oxygen. These components are individually harmless but, when combined, generate potent reactive oxygen species (ROS), mainly singlet oxygen. ROS can be produced both inside and outside the target cells depending on the localization of the photosensitizer. Moreover, they are strong oxidizing and cytotoxic agents, leading to cell death. ROS have multiple cellular targets, including the cell membrane, membrane proteins, cytoplasmic enzymes, mitochondria, endoplasmic reticulum, nuclear material, and other cellular components. These multiple targets hinder the development of resistance mechanisms by tumor cells and microorganisms [4,5,6].

PDT exerts significant effects not only on target cells but also on the surrounding vasculature and immune system. Upon activation of a photosensitizer by light of a specific wavelength in the presence of molecular oxygen, ROS are generated, leading to oxidative damage in nearby biological structures. In the vascular compartment, PDT can induce endothelial cell injury, platelet aggregation, vasoconstriction, and increased vascular permeability, ultimately resulting in vascular shutdown and reduced blood supply to the treated tissue. This vascular damage contributes to ischemia and enhances the therapeutic efficacy against infected or diseased cells. In parallel, PDT has been shown to stimulate both innate and adaptive immune responses. The oxidative stress and cellular damage induced by PDT promote the release of damage-associated molecular patterns, pro-inflammatory cytokines (IL-1β, TNF-α, and IL-6), and tumor- or pathogen-associated antigens, which recruit and activate immune cells such as neutrophils, macrophages, and dendritic cells. These events facilitate antigen presentation and the activation of cytotoxic T lymphocytes, contributing to systemic immune responses and immunological memory. Consequently, PDT is considered not only a local cytotoxic treatment but also an immunomodulatory approach capable of enhancing host defense mechanisms against infections and tumors [1,2,3,4,5,6].

Phthalocyanines (PCs) are among the most widely used photosensitizers for evaluating antitumoral and antimicrobial photobiological activities in PDT [7,8,9]. PCs exhibit high singlet oxygen generation upon light activation and low cytotoxicity in the dark, making them suitable for PDT applications [4,5,6,7]. Aluminum phthalocyanine chloride (Al-Pc-Cl) is a phthalocyanine with appropriate properties for PDT. However, Al-Pc-Cl shows low solubility in aqueous media and instability due to the formation of photobiologically inactive dimers. Therefore, encapsulation of Al-Pc-Cl in a nanocarrier would preserve its photobiological properties, enable transport to the target tissue, and provide sustained release, thereby promoting PDT in vivo [8,10].

Pharmaceutical nanotechnology has significantly advanced PDT because nanocarriers such as nanoparticles (NPs) [9], nanoemulsions [8], liposomes, and niosomes [10] are capable of encapsulating, stabilizing, transporting, and prolonging the release of photosensitizers, thus improving their antitumoral and antimicrobial photobiological activities and enhancing PDT outcomes [8,9,10,11].

Polymeric nanoparticles (PNPs) are nanoscale drug-delivery systems typically ranging from 10 to 1000 nm, composed of natural or synthetic biodegradable polymers such as poly(lactic-co-glycolic acid) (PLGA), polycaprolactone (PCL), chitosan, or polyethylene glycol (PEG). Polymeric nanoparticles are being widely explored in pharmaceutical and biomedical applications due to their ability to enhance drug stability, improve bioavailability, enable controlled or targeted release, and reduce systemic toxicity. Their physicochemical characterization typically involves the determination of particle size and polydispersity index (PdI) by dynamic light scattering (DLS), surface charge (zeta potential) by electrophoretic mobility analysis, and morphology by scanning electron microscopy (SEM) or transmission electron microscopy (TEM) drug loading, encapsulation efficiency, and in vitro release studies. PNPs have been used as nanocarriers for photosensitizers while maintaining their photobiological activity. One of the most commonly used polymers for nanoparticle preparation is polycaprolactone (PCL). Due to its biodegradability and non-toxicity, PCL has been extensively investigated for drug and photosensitizer encapsulation [8,9,10,11].

Magnetic nanoparticles (MagNPs), typically composed of magnetite (Fe_3_O_4_) or maghemite (γ-Fe_2_O_3_), are a class of nanomaterials widely investigated for biomedical applications due to their superparamagnetic behavior, biocompatibility, and ease of surface functionalization. These nanoparticles generally range from 5 to 100 nm in diameter and can be synthesized by methods such as coprecipitation, thermal decomposition, hydrothermal synthesis, and microemulsion techniques. Their magnetic properties allow external magnetic fields to control their movement and localization, making them particularly useful in drug delivery, magnetic resonance imaging (MRI) contrast enhancement, hyperthermia therapy, and biosensing. Additionally, surface modification with polymers, surfactants, or biomolecules improves their colloidal stability, biocompatibility, and targeting capability in biological systems. MagNPs have been co-encapsulated with drugs in nanocarriers to enable targeting to tissues through the application of an external magnetic field [12]. MagNPs can be easily produced through chemical coprecipitation reactions using iron salts. The aqueous coprecipitation method is the simplest and most practical approach for preparing iron-oxide magnetic nanoparticles. Iron-oxide-based MagNPs should be coated with oleic acid to stabilize the system and subsequently encapsulated in PNPs to improve biocompatibility and stability in aqueous media [12,13].

To the best of our knowledge, there is no literature report of polymeric nanoparticles containing aluminum phthalocyanine chloride (Al-Pc-Cl) and iron-oxide magnetic nanoparticles. Based on the challenges associated with treatments of fungal infections with conventional antifungals and MRSA infections with vancomycin as well as overwhelming challenges associated with cancer chemotherapy, we hypothesize that PDT with polymeric nanoparticles containing aluminum phthalocyanine chloride (Al-Pc-Cl) and iron-oxide magnetic nanoparticles would improve treatment of cancer and resistant fungal and bacterial infections such as recurrent vulvovaginal candidiasis and MRSA infections.

In this study, we aim to develop a polymeric nanocarrier containing Al-Pc-Cl and MagNPs, followed by physicochemical characterization and evaluation of the antitumor and antimicrobial photobiological activities of the developed formulations. MagNPs were prepared by the coprecipitation of Fe^3+^ and Fe^2+^ ions under alkaline pH conditions [12,13]. PNPs containing the photosensitizer (Al-Pc-Cl) and MagNPs were prepared using the emulsification–solvent evaporation method. The nanosystems were characterized in terms of process yield, photosensitizer content, size distribution, polydispersity index (PdI), and morphology by transmission electron microscopy (TEM). An in vitro release study was conducted to ascertain if the nanoparticles would control Al-Pc-Cl release. The developed delivery systems were also assessed for antitumoral and antimicrobial photobiological activities.

## 2. Materials and Methods

### 2.1. Synthesis of Magnetic Nanoparticles (MagNPs)

For the synthesis of MagNPs, anhydrous ferric chloride (FeCl_3_) and ferrous chloride tetrahydrate (FeCl_2_·4H_2_O) were purchased from Merck (São Paulo, Brazil). MagNPs were synthesized by coprecipitation of an aqueous Fe^3+^/Fe^2+^ solution using ammonium hydroxide (Merck, São Paulo, Brazil). The pH was adjusted to 10 to promote MagNP precipitation. Briefly, 21 g of FeCl_3_ and 17.3 g of FeCl_2_·4H_2_O were dissolved in 50 mL of distilled water. The reaction vessel was kept under a nitrogen (N_2_) atmosphere to prevent reactions of iron ions with oxygen. Subsequently, 40 mL of ammonium hydroxide was added to induce MagNP precipitation and pH of the reaction was controlled using pH-indicator strips (Merck, São Paulo, Brazil). A quantity of 20 mL of oleic acid (Merck, São Paulo, Brazil) was added in liquid form at 80 °C to coat the magnetic iron oxide nanoparticles and stabilize the suspension. The mixture was heated at 80 °C for 30 min to complete the oleic-acid-coating process. The suspension was washed with distilled water to remove ammonium hydroxide, followed by acetone to eliminate excess oleic acid, and again with water to remove residual acetone. Finally, the MagNPs were lyophilized and stored in a desiccator for subsequent encapsulation into PCL nanoparticles [12,13].

### 2.2. Development of PNPs Containing PS(Al-Pc-Cl)

Aluminum phthalocyanine chloride (Al-Pc-Cl) (Merck, São Paulo, Brazil; 95% purity; MW = 574.96 g mol^−1^) was dissolved in dimethyl sulfoxide (DMSO) (Tédia, São Paulo, Brazil) to prepare a standard solution at a concentration of 0.5 mg mL^−1^. Polymeric nanoparticles (PNPs) were produced using the emulsion–solvent evaporation method [14]. The organic phase consisted of poly(ε-caprolactone) (PCL; MW = 42,500–65,000; Sigma-Aldrich, St. Louis, MO, USA) (150 mg), Al-Pc-Cl (300 µg), and dichloromethane (DCM) (Tédia, São Paulo, Brazil) (5 mL). The aqueous phase comprised 1% (*w*/*w*) poly(vinyl alcohol) (PVA; 87–89% hydrolyzed; MW = 13,000–23,000; Sigma-Aldrich, St. Louis, MO, USA) (50 mL) in distilled water. PNPs–PS(Al-Pc-Cl) were prepared by adding the organic phase to the aqueous phase under homogenization using an ultrasonic processor (UP100H, Hielscher^®^, Schwabach, Germany) operating at Cycle 1 and 100% amplitude for 5 min in an ice bath at 5 °C. The organic solvent (DCM) was removed using a rotary evaporator (IKA^®^ RV 10, São Paulo, Brazil) under reduced pressure at 40 °C for 3 h to allow nanoparticle formation. The suspension was purified by three consecutive centrifugation cycles at 20,000× *g* for 20 min (Avanti J-25; Beckman Coulter, San Francisco, CA, USA), with resuspension in ultrapure water between cycles to remove surfactant and non-encapsulated drug. Finally, the nanoparticle suspension was transferred to glass vials, rapidly frozen in liquid nitrogen, and lyophilized using a bench-top freeze-drying system (FreeZone 4.5-L; Labconco, Kansas City, MO, USA).

### 2.3. Characterization of Nanocarriers

#### 2.3.1. Process Yield (Y%)

The mass of lyophilized particles was weighed to calculate the process yield (Y) using Equation (1):

(1) $$Y(\%)=\frac{m1}{m2}\times 100$$

where Y (%) is the process yield, *m*1 is the mass of particles recovered after lyophilization, and *m*2 is the total mass of polymer plus drug used in the formulation. The process yield was determined after triplicate analysis (n = 3).

#### 2.3.2. Encapsulation Efficiency (EE%)

The encapsulation efficiency of the drug (EE%) in PNPs was determined by correlating the total amount of drug recovered with the total amount initially added during nanoparticle preparation. For this purpose, the drug was extracted from the PNPs. For Al-Pc-Cl extraction, 10 mg of lyophilized PNPs were added to 10 mL of acetone (Tédia, São Paulo, Brazil) and maintained at 60 °C for approximately 10 min. Heating was performed using a beaker placed on a heating plate (IKA^®^, C-MAG, São Paulo, Brazil) to dissolve the PNPs and extract Al-Pc-Cl, yielding a theoretical concentration of 100 ng/mL. The Al-Pc-Cl concentration was determined using an analytical calibration curve obtained by a validated spectrophotometric method with a spectrophotometer (Jasco V-630, Tokyo, Japan). Encapsulation efficiency was calculated according to Equation (2):

(2) $$\mathrm{EE}(\%)=\frac{m1}{m2}\times 100$$

where EE (%) is the encapsulation efficiency, *m*1 is the mass of drug obtained from Al-Pc-Cl-loaded PNPs, and *m*2 is the theoretical drug mass used in the formulation.

#### 2.3.3. Drug Content (DC)

Based on the encapsulation efficiency results, the drug loading (µg of Al-Pc-Cl per mg of PNPs) in the nanocarriers was calculated. Drug content represents the relationship between the amount of encapsulated drug and the process yield. The Al-Pc-Cl content in PNPs was calculated using the mean values of process yield (R%) and encapsulation efficiency (EE%). The drug content was determined according to Equation (3):

(3) $$\mathrm{DC}\;(\mu g/\mathrm{mg})\;=\frac{m1}{m2}$$

where T is the Al-Pc-Cl content (µg of Pc per mg of PNPs), *m*1 is the Pc mass determined from EE (%) in µg within the nanoparticles, and *m*2 is the PNP mass obtained from the process yield (R%) in mg.

#### 2.3.4. Size, Polydispersity Index, Zeta Potential, and Morphology

##### Hydrodynamic Diameter and Polydispersity Index

The mean diameter (nm) and polydispersity index (PdI) of the PNPs were measured using a Zetasizer Nano^®^ S90 (Malvern Instruments, Worcestershire, UK) by dynamic light scattering (DLS). An aliquot of 20 µL of the nanoparticle suspension was diluted in 1 mL of distilled water prior to analysis. Size distribution and PdI were determined using the instrument’s light-scattering module.

##### Zeta Potential

Zeta potential measurements were performed using the same instrument (Zetasizer Nano^®^ S90, Malvern Instruments, UK) for PNPs, PNPs-PS, MagNPs, and PNPs-PS-MagNPs. Samples were diluted (1:50) in TRIS buffer (pH 6.8). All analyses were performed in triplicate, and results were expressed as mean ± standard deviation.

##### Morphological Analysis (TEM)

The PNP suspension was diluted with water (1:1) and deposited onto microscopy grids (Formvar, 200 mesh, copper; dpUnion, São Paulo, Brazil). After 1 min of sample adsorption, excess liquid was removed using filter paper. The grids were dried in a desiccator for 2 h and analyzed by transmission electron microscopy (TEM) (Tecnai Spirit 7800, Hitachi High-Tech Corporation, Tokyo, Japan).

#### 2.3.5. Quantification of the Photosensitizer

Quantitative analysis of Al-Pc-Cl encapsulated in PNPs was performed by spectrophotometry using a Jasco^®^ V-630 spectrophotometer (Tokyo, Japan), following the methodology described in the literature (2022) [10,14]. Al-Pc-Cl was dissolved in ethanol (Tédia, São Paulo, Brazil) to prepare a 2.5 µg/mL solution. A spectral scan from 200 to 800 nm was conducted to determine the maximum absorption wavelength (λ_max_). The λ_max_ of Al-Pc-Cl in ethanol was 670 nm, consistent with literature data [10,15]. This solution was subsequently diluted with ethanol to concentrations ranging from 0.04 to 0.4 µg/mL to construct the analytical Beer-Lamberts calibration curve. The curve showed excellent linearity (r > 0.999). Lyophilized Al-Pc-Cl-loaded PNPs were dissolved in acetone (Tédia, São Paulo, Brazil) at 60 °C using a heating plate with magnetic stirring (IKA^®^, C-MAG, São Paulo, Brazil). The solution was centrifuged at 20,000× *g* for 20 min (Avanti J-25, Beckman Coulter, San Francisco, CA, USA) to remove solid residues. The supernatant was diluted in ethanol for quantification of the encapsulated photosensitizer. Absorbance was measured spectrophotometrically, and the PS content (µg of PS per mg of PNPs) was calculated using the analytical curve (0.04–0.4 µg/mL in ethanol, λ_max_ = 670 nm, r > 0.999).

#### 2.3.6. Thermogravimetric Analysis (TGA)

Thermogravimetric analysis (TGA) measures sample mass loss as a function of increasing temperature. The data were presented as temperature versus percentage mass loss. Samples were weighed, placed in the instrument pan, and heated at a rate of 25 °C/min over a temperature range of 30 °C to 700 °C using a thermogravimetric analyzer (TGA Q500 V67 Build 203, New Castle, DE, USA). TGA was used to determine the amount of magnetic nanoparticles (MagNPs) encapsulated within the polymeric nanoparticles in the sample PNPs-PS-MagNPs.

### 2.4. In Vitro Release Study and Kinetic Analysis

After the development of PNPs-PS and PNPs-PS-MagNPs, in vitro release studies were performed to evaluate the ability of the nanocarrier to release the encapsulated PS as a function of time. A volume of 60 mL of receptor medium consisting of isotonic phosphate-buffered saline (PBS, pH 7.4) containing 0.1% sodium n-dodecyl sulfate (SDS) was transferred to a 100 mL beaker. The beaker was immersed in a thermostatic bath and maintained at 37 °C to ensure thermal equilibrium. A mass of 50 mg of PNPs-PS or PNPs-PS-MagNPs was added to the receptor solution. The resulting suspension was continuously stirred at 100 rpm using a magnetic bar on a stirring plate (MAG-Mult 15, Marte, São Paulo, Brazil). At predetermined time intervals (0, 2, 4, 6, 12, 24, 36, 48, 72, 96, and 120 h), 3 mL aliquots were withdrawn and centrifuged at 10,000× *g* for 10 min using a centrifuge (Avanti J-25, Beckman Coulter, San Francisco, CA, USA). The particle precipitate was separated from the supernatant. The absorbance of the released PS in the receptor medium was measured at 670 nm using a spectrophotometer (V-630, Jasco, Tokyo, Japan), and the concentration was calculated from the analytical calibration curve of Al-Pc-Cl obtained by spectrophotometry. Immediately after measurement, the supernatant and precipitate were recombined, homogenized to redisperse the nanoparticles, and returned to the beaker to continue the release experiment. The assay was performed in triplicate (n = 3). Absorbance values were correlated with concentration (µg/mL), and linear regression was used to obtain the equation of the analytical curve. The calculated concentrations were converted into mass values and used to construct the in vitro release profile as a function of time. Mathematical models were applied to describe the drug release kinetics from the delivery system. The most commonly used models that best describe drug release phenomena are the Higuchi, zero-order, and first-order models.

The zero-order kinetics model applies to dosage forms that do not undergo disintegration and release a constant amount of drug per unit time, representing an ideal system for prolonged pharmacological action due to its slow and controlled release [8,10]. This model is expressed by Equation (4):

(4) Q_t_ = Q_0_ + K_0_t

where Q_t_ is the amount of drug released at time t; Q_0_ is the initial amount of drug in solution; and K_0_ is the zero-order kinetic constant.

The first-order kinetics model is characteristic of dosage forms in which drug release is proportional to the amount of drug remaining in the system, resulting in a decrease in the amount released per unit time. This model is expressed by Equation (5):

(5) ln Q_t_ = ln Q_0_ + K_t_t

where Q_t_ is the amount of drug released at time t; Q_0_ is the initial amount of drug in solution; and K_t_ is the first-order release constant.

The Higuchi model, which is based on Fick’s law, describes drug release from solid matrices governed by diffusion and is represented by Equation (6):

(6) Q_t_ = K_H_ + √ t

where Q_t_ is the amount of drug released at time t; and K_H_ is the Higuchi release constant.

After applying the kinetic models, linear regression analysis was used to determine the equation of the line and the correlation coefficient (r). The value of “r” closest to 1 indicated the mathematical model that best fit the drug-release kinetics of the nanoparticles.

### 2.5. Photobiological Antitumoral and Antimicrobial Activity Studies

#### 2.5.1. Photobiological Antitumoral Activity

A549 cells (human lung adenocarcinoma cell line, BCRJ Code 0033, Rio de Janeiro Cell Bank) were thawed at 37 °C and transferred to a 25 mL culture flask containing 7 mL of DMEM medium (Merck, São Paulo, Brazil) supplemented with 10% fetal bovine serum. The culture flask was maintained at 37 °C in a humidified atmosphere with 5% CO_2_ for 48 h prior to the first passage. Cell passaging involved removal of the DMEM medium, washing with PBS, and addition of 1 mL of Trypsin solution containing HEPES (10 mM) (Merck, São Paulo, Brazil) and EDTA (0.2 mM) (Merck, São Paulo, Brazil) to detach the adherent cells. The cell suspension was centrifuged to remove the solution and resuspended in 2 mL of DMEM. A 25 µL aliquot was used for cell counting. Cell concentration was determined using the trypan blue exclusion assay (Merck, São Paulo, Brazil) with a Neubauer chamber. Aliquots containing 1 × 10^5^ cells/mL were seeded into 96-well plates with 250 µL of culture medium per well. After 24 h incubation to allow cell adhesion, cytotoxicity (dark assay) and photobiological antitumoral activity were evaluated according to protocols established by our research group [16]. For both cytotoxicity and photobiological assays, Al-Pc-Cl (PS, photosensitizer) encapsulated in nanoparticles was used at a concentration of 10 µg/mL. PNPs-PS and PNPs-PS-MagNPs were incubated with cells in DMEM medium for 24 h at 37 °C. After incubation time, the medium was then removed, cells were washed with PBS, and fresh DMEM was added. Cells assigned to the photobiological antitumoral assay were irradiated with red light (λ = 660 nm) at a dose of 30 J/cm^2^ (Photon Laser I, DMC) (Supplementary Materials: Figure S1) and then incubated again at 37 °C for 24 h. Experiments were performed in sextuplicate (n = 6). Cell viability was determined by adding 50 µL of MTT solution (1 mg/mL, Merck, São Paulo, Brazil) to each well, followed by 3 h incubation. Formazan crystals were dissolved in DMSO (Tédia, Rio de Janeiro, Brazil), and absorbance was measured at 595 nm using a microplate spectrophotometer (SpectraMax 340 Microplate Reader, Molecular Devices^®^, San Jose, CA, USA). The MTT assay quantifies viable cells with intact mitochondrial activity. The key component is the dye 3-(4,5-dimethylthiazol-2-yl)-2,5-diphenyl tetrazolium bromide (MTT), which is reduced by mitochondrial dehydrogenases of viable cells to form insoluble purple formazan crystals. After dissolution in DMSO, absorbance values were measured to determine cell viability. For each sample, cell viability was calculated and expressed as a percentage relative to untreated control cells (considered 100% viability). Comparison between the mean optical density of untreated and treated cells 24 h after irradiation allowed evaluation of the photobiological antitumoral activity.

The experiments were performed in sextuplicate (n = 6). In the antitumor photobiological study, statistical analysis comparing cell viability between irradiated and non-irradiated samples was conducted using an unpaired Student’s *t*-test (GraphPad Prism version 5.0 for Windows, GraphPad Software, San Diego, CA, USA). Additionally, differences in cell viability among the irradiated groups were analyzed using one-way analysis of variance (ANOVA) followed by Tukey’s multiple comparison test. Differences were considered statistically significant when *p* < 0.05.

#### 2.5.2. Antimicrobial Photobiological Activity and IC_50_ Determination

The antimicrobial photobiological activity of PNPs-PS, PNPs-PS-MagNPs, and PS (free Al-Pc-Cl) was evaluated by determining the IC_50_ against clinically relevant pathogenic microorganisms, including strains resistant to conventional drugs. Microbial strains were provided by the Oral Microbiology Laboratory (UFRJ, Rio de Janeiro, Brazil). Analyses were performed both in the dark (without light irradiation) and under light exposure for PS activation.

Reference strains obtained from the American Type Culture Collection (ATCC) were used: *Staphylococcus aureus* ATCC 43300 (methicillin-resistant, MRSA) and *Candida albicans* ATCC 10231. MRSA was cultured on Mueller–Hinton agar, following the recommendations of the Clinical and Laboratory Standards Institute (CLSI) [17]. The yeasts *Candida albicans* and *Candida auris* were cultured in Sabouraud Dextrose Broth (SDB) and maintained on Sabouraud Dextrose Agar (SDA), according to the methodology described in earlier study [18]. Bacterial cultures were incubated at 37 °C for 18–24 h under aerobic conditions, while fungal cultures were incubated at 30 °C for 24–48 h. Prior to the assays, microorganisms were resuspended in sterile saline solution (0.9% NaCl) and adjusted to a turbidity equivalent to the 0.5 McFarland standard, corresponding to approximately 1 × 10^8^ CFU/mL for bacteria and 1 × 10^6^ CFU/mL for yeasts.

Microbial suspensions were inoculated into 96-well plates containing 200 µL per well of different PS (Al-Pc-Cl) concentrations, yielding final concentrations of 30, 15, 7.5, 3.75, 1.875, 0.9375, 0.468, and 0.234 µg PS/mL of culture medium. As a positive control, microbial growth in culture medium without antibiotic addition was used, whereas the negative control consisted of sterility tests containing only culture medium. All experiments were performed in sextuplicate (n = 6 determinations).

The non-irradiated group was used as a control to evaluate the dark antimicrobial activity of PNPs-PS, PNPs-PS-MagNPs, and PS. The irradiated group received a standard dose of visible red light (λ_max_ = 660 nm) with an irradiation of 30 J/cm^2^ using a low-intensity therapeutic laser (Photon Lase I, DMC^®,^ São Carlos, Brazil) (Supplementary Materials: Figure S1) to assess the photobiological activity of the formulations. The light dose used in the antimicrobial photobiological assays was standardized in previous studies by our research group [8,10].

After irradiation, the prepared plates were incubated for 24 hours for microbial growth according to the established protocol by our research group [8], and cell viability was subsequently evaluated using the resazurin assay. This colorimetric assay is based on the metabolic capacity of viable cells to reduce resazurin to resorufin. Only viable cells retain the ability to reduce resazurin (purple/blue) to resorufin (pink), resulting in changes in absorption and fluorescence properties. After the incubation period of 24 hours, plates were stained with 30 µL of 0.01% resazurin solution (Sigma-Aldrich^®^, São Paulo, Brazil). Resazurin (7-hydroxy-3H-phenoxazin-3-one-10-oxide) is a blue dye with weak fluorescence that is reduced to resorufin, a pink dye exhibiting strong fluorescence in the red spectrum [19].

The IC_50_, defined as the inhibitory concentration required to inactivate 50% of the microorganisms, was determined by regression analysis using GraphPad Prism 5.0 software (GraphPad Software, CA, USA). IC_50_ values were calculated based on the cytotoxicity curves generated in the experiment. Microplates were read at 590 nm using a microplate spectrophotometer (SpectraMax 340 Microplate Reader, Molecular Devices^®^, San Jose, CA, USA). The obtained values were used to construct graphs expressing the percentage of microbial viability.

The experiments were performed in sextuplicate (n = 6). In the antimicrobial photobiological study, statistical analysis comparing IC_50_ between “irradiated and non-irradiated samples” was conducted using an unpaired Student’s *t*-test (GraphPad Prism version 5.0 for Windows, GraphPad Software, CA, USA). Additionally, differences in IC_50_ among the “irradiated groups” or “irradiated groups including the reference drug” were analyzed using one-way analysis of variance (ANOVA) followed by Tukey’s multiple comparison test. Differences were considered statistically significant when *p* < 0.05.

## 3. Results and Discussion

### 3.1. Preparation and Macroscopic Analysis

**Figure 1:**
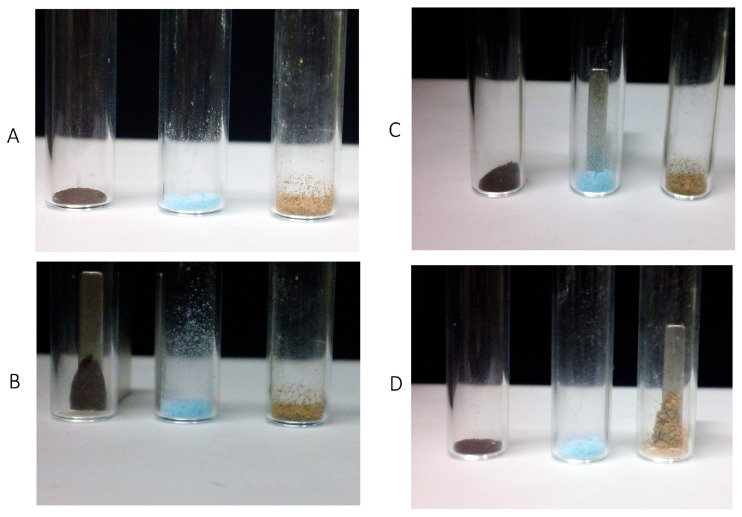
(**A**) MagNPs (black), PNPs-PS (blue) and PNPs-PS-MagNPs (light brown); (**B**) MagNPs (black) being attracted by the magnet; (**C**) PNPs-PS (blue) not being attracted by the magnet; (**D**) PNPs-PS-MagNPs (light brown) being attracted by the magnet.

The MagNPs, PNPs-AlPc-Cl, and PNPs-AlPc-Cl-MagNPs were successfully produced. Figure 1 shows images of the lyophilized powder obtained after water removal, which enabled sample stabilization and storage in a desiccator. Figure 1A presents (from left to right) the MagNPs (black), PNPs-PS (blue), and PNPs-PS-MagNPs (light brown). Figure 1B, Figure 1C, and Figure 1D show a magnet being placed near MagNPs (black), PNPs-PS (blue), and PNPs-PS-MagNPs (light brown), respectively. As shown in Figure 1B,D, the magnet was able to attract MagNPs and PNPs-PS-MagNPs, confirming their magnetic responsiveness. In contrast, the material shown in Figure 1C (PNPs-PS) was not attracted because it does not contain magnetic nanoparticles. These results demonstrate that the simultaneous encapsulation of PS and MagNPs into the polymeric nanoparticles was successful and that the encapsulation process did not alter the magnetic properties.

### 3.2. Particle Size, PdI, and Zeta Potential

PNPs were prepared using the emulsion–solvent evaporation method, and the size, PdI, and zeta potential values are presented in Table 1. MagNPs exhibited an average nanoscale size of 33.6 nm with a PdI lower than 0.3, indicating a monodisperse system [10,14]. PNPs and PNPs-PS showed sizes below 200 nm, demonstrating that encapsulation of the photosensitizer (Al-Pc-Cl) did not significantly alter the particle size. Moreover, PdI values remained below 0.1, indicating a narrow size distribution and high monodispersity [8,10,14]. For PNPs-PS-MagNPs, the incorporation of MagNPs influenced the nanocarrier size, increasing it to 333.56 nm; however, the PdI remained below 0.1, still indicating a monodisperse system [10,14]. NPs-PS-MagNPs are larger than PNPs-PS because they contain multiple MagNPs in their structure. The size increased from 186.9 nm (PNPs-PS) to 333.56 nm (PNPs-PS-MagNPs) keeping a PdI of 0.1. NPs-PS-MagNPs do not form clusters, as shown using transmission electron microscopy (TEM). All samples exhibited negative zeta potential values. Charged nanoparticles generally present higher stability in dispersed systems due to electrostatic repulsion, which prevents aggregation. The highly negative value observed for MagNPs can be attributed to the oleic-acid-based surface coating.

### 3.3. Morph3.3 Morphological Analysis by TEM

**Figure 2:**
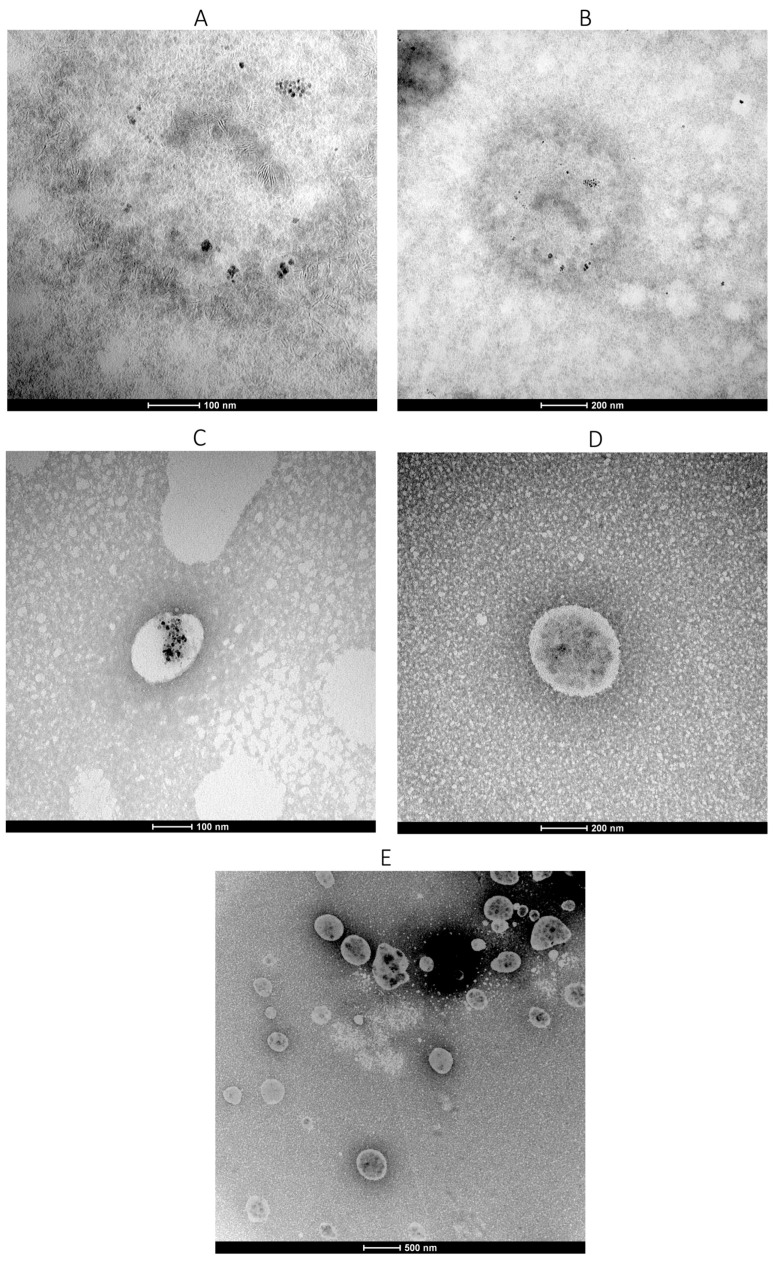
TEM of MagNPs (**A**), MagNPs at lower magnification (**B**), PNPs-PS-MagNPs (**C**) isolated, PNPs-PS-MagNPs isolated at lower magnification (**D**) and Panoramic image of PNPs-PS-MagNPs (**E**).

**Figure 3:**
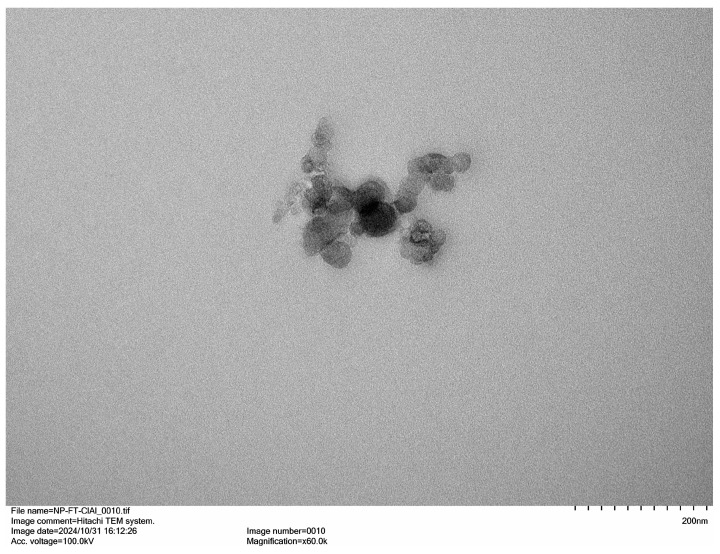
PNP-PS images obtained by TEM.

Morphological analysis was performed by TEM. Figure 2A and Figure 2B show MagNPs at higher and lower magnifications, respectively. The nanoparticles appear mostly isolated, although some agglomeration was observed, attributable to water removal during sample preparation, which reduced interparticle distance. Figure 2C–E show PNPs-PS-MagNPs at different magnifications. In Figure 2C, MagNPs (dark spots) can be observed encapsulated within the polymeric matrix. Figure 2E presents a panoramic view, whereas Figure 2D shows an isolated nanoparticle. The presence of dark spots inside the PNPs confirms successful encapsulation of MagNPs within the polymeric nanocarrier. Notably, PNPs-PS-MagNPs did not form aggregates after water removal and displayed a spherical morphology. TEM shows that NPs-PS-MagNPs do not form agglomerates. Furthermore, MagNPs are not magnets; they do not attract each other, but they respond to the action of a magnetic field from a magnet. Additional images are provided in the Supplementary Materials (Figure S2). Figure 3 shows TEM images of PNPs-PS. Although some aggregation occurred, the nanoparticles maintained a spherical shape. This aggregation is attributed to elimination of the aqueous dispersant during sample preparation prior to analysis.

### 3.4. Yield (R%), Encapsulation Efficiency (EE%), and Drug Loading

The spectrophotometric profile of the PS showed a maximum absorption wavelength at 670 nm in ethanol. The analytical curve was constructed over the concentration range of 0.04–0.40 μg/mL, yielding a linear correlation coefficient (r) greater than 0.99 (Supplementary Materials, Figure S3). After dissolving the polymeric nanoparticles in hot acetone followed by dilution in ethanol, the PS concentration was determined using the calibration curve. Subsequently, the solution concentration, mass of encapsulated PS, encapsulation efficiency (EE%), and drug loading were calculated and the results are shown in Table 2. PNPs-PS presented a process yield of 75% and an encapsulation efficiency of 95%. PNPs-PS-MagNPs showed a higher yield; however, encapsulation efficiency decreased to 87%, likely due to partial PS loss during the encapsulation process. Drug content values were 2.92 and 2.35 μg Al-Pc-Cl/mg PNPs for PNPs-PS and PNPs-PS-MagNPs, respectively.

### 3.5. Thermogravimetric Analysis (TGA)

The amount of MagNPs encapsulated in the PNPs was determined by TGA (Supplementary Materials, Figure S4). PNPs-PS exhibited approximately 95% mass loss attributed to polymer thermal degradation between 300 and 400 °C, consistent with literature reports [20,21]. In contrast, PNPs-PS-MagNPs showed a lower mass loss (86%) between 300 and 425 °C, with a residual mass of 13%. MagNPs displayed thermal stability within the investigated temperature range and did not undergo degradation. Therefore, TGA analysis indicates that approximately 13% of MagNPs were encapsulated within the polymeric nanoparticles. A similar study using PMMA-based PNPs reported magnetic material encapsulation of approximately 11% [13]. Thermogravimetric analysis (TGA) is an appropriate technique for determining the amount of encapsulated iron oxide nanoparticles. During the heating process, the organic polymeric matrix undergoes thermal decomposition and is completely degraded at temperatures around 300 to 425 °C. Consequently, the residual mass observed at this temperature corresponds to the inorganic nanoparticle content, enabling the quantification of the encapsulated iron oxide within the polymeric system.

### 3.6. In Vitro Release Profile and Kinetics

**Figure 4:**
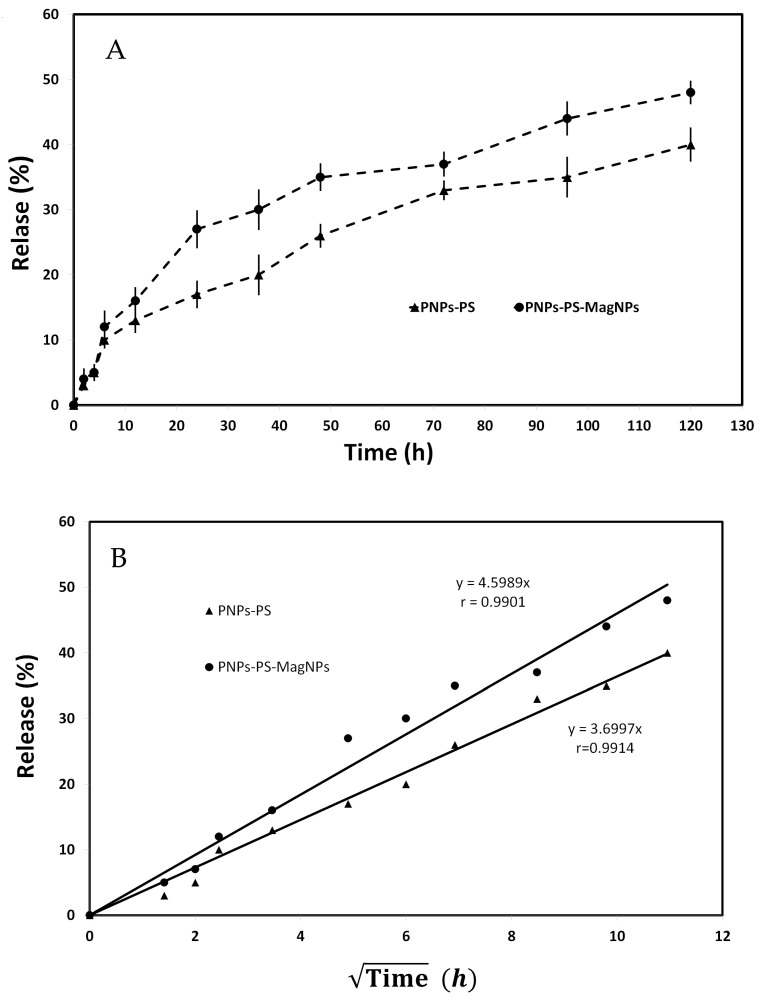
In vitro release profile of the PS in the receptor medium (mean ± S.D. of n = 3 determinations) (**A**); kinetic of Higuchi’s model (mean of n = 3 determinations) (**B**).

The PS release profile is presented in Figure 4A. Drug release was slow and sustained over 120 h, with maximum release values of 41% and 49% for PNPs-PS and PNPs-PS-MagNPs, respectively. The remaining fraction of the photosensitizer remained encapsulated within the nanoparticle matrix, indicating a sustained-release profile. This unreleased fraction may continue to be gradually liberated over period exceeding 120 h, suggesting prolonged drug release. Drug release from nanoparticles is a multifactorial process that may occur through different mechanisms, individually or simultaneously, depending on the physicochemical characteristics of the polymeric matrix and environmental conditions. The main mechanisms include the following: drug desorption from the nanoparticle surface, leading to an initial burst release; diffusion through hydrophilic channels or pores within the polymer matrix; progressive matrix erosion causing structural disruption and formation of additional release pathways; a synergistic combination of diffusion and polymer degradation (erosion), promoting sustained release. The predominance of each mechanism depends on the type of delivery system, polymer nature (hydrophilic or lipophilic), presence of functionalizing agents, and physiological microenvironmental conditions (pH, ionic strength, receptor volume), directly influencing release kinetics and therapeutic performance [14,22,23]. Zero-order, Higuchi, and first-order kinetic models were applied to the release data to determine the best fit. Model selection was based on the linear correlation coefficient (r) obtained from regression analysis. The Higuchi model showed the highest correlation coefficients and best described the release behavior (Table 3), indicating the presence of a diffusion-controlled mechanism.

The Higuchi plot derived from the in vitro release data is shown in Figure 4B. Although the release profiles of PNPs-PS and PNPs-PS-MagNPs differed quantitatively, both followed Higuchi kinetics, confirming diffusion as the primary mechanism governing PS release from the polymeric nanoparticles.

### 3.7. Evaluation of Antitumoral Photobiological Activity

**Figure 5:**
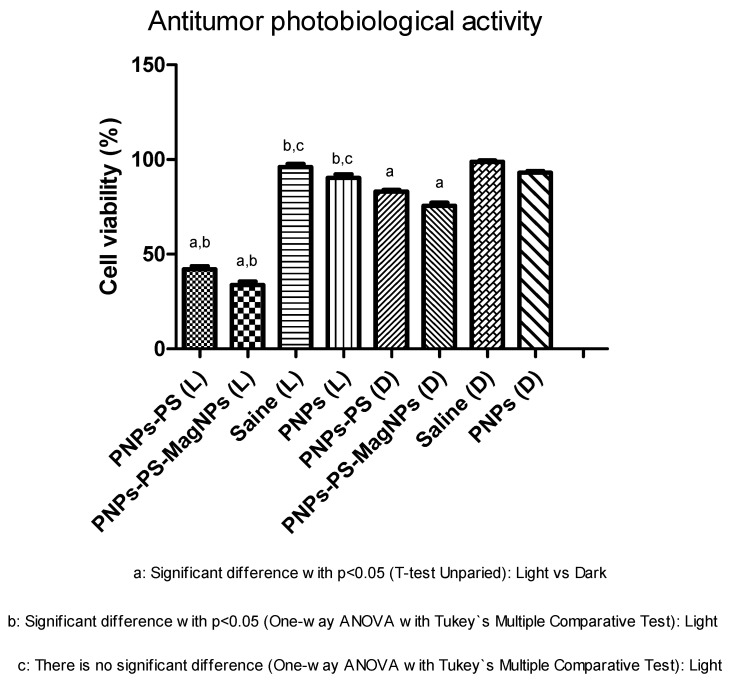
Antitumor photobiological activity and cytotoxicity (L) = sample irradiated with light and (D) = without irradiation. Each value represents the mean ± S.D. of six independent determinations (n = 6). Cell viability values (%) obtained for PNPs, PNPs-PS, PNPs-PS-MagNPs in A549 cell line under irradiation (λ = 660 nm; 30 J/cm^2^). Note: “a” indicates a statistically significant difference (*p* < 0.05) between irradiated and non-irradiated samples (unpaired *t*-test); “b” indicates a statistically significant difference (*p* < 0.05) between irradiated samples (one-way ANOVA followed by Tukey’s multiple comparisons test); “c” indicates that there is no significant difference between irradiated samples (one-way ANOVA followed by Tukey’s multiple comparisons test).

The assay exposed the human lung cancer cell line A549 to the formulations—PNPs without photosensitizer, PNPs-PS, and PNPs-PS-MagNPs. The results are presented in Table 4 and Figure 5. The toxicity of the delivery systems toward the cell culture in the absence of light was evaluated using the MTT assay associated with a spectrophotometric method, which detects damage to mitochondrial activity and estimates the number of viable cells. According to ISO 10993-5:2009, the MTT assay establishes that a material is considered to have cytotoxic potential when cell viability decreases below 70% relative to the blank control. Conversely, when cell viability remains above this threshold, the material is classified as non-cytotoxic [24,25,26]. PNPs without photosensitizer and saline solution showed cell viabilities of 98.72 ± 2.21% and 92.88 ± 2.72%, respectively. PNPs-PS and PNPs-PS-MagNPs reduced cell viability to 82.98 ± 3.11% and 75.51 ± 2.85%, respectively, but were not considered cytotoxic according to ISO 10993-5:2009 [24,25,26]. The absence of cytotoxicity for PNPs-PS and PNPs-PS-MagNPs is an important finding because it enables subsequent evaluation of antitumoral photobiological activity. Upon light irradiation, both formulations promoted significant photobiological antitumoral effects, reducing cell viability to 42.05 ± 2.17% and 34.11 ± 1.75%, respectively, with statistically significant differences compared with PNPs without photosensitizer and saline. Thus, PNPs-PS and PNPs-PS-MagNPs exhibited significant antitumoral photobiological activity (Table 4).

### 3.8. Evaluation of Antimicrobial Photobiological Activity

Antimicrobial photobiological activity was assessed by determining the concentration of photosensitizer (PS) required to reduce cell viability by 50% (IC_50_) for irradiated and non-irradiated groups against different microbial strains. Table 5 presents the IC_50_ values (µg/mL) obtained for irradiated and non-irradiated conditions against methicillin-resistant *Staphylococcus aureus* (MRSA) and *Candida albicans*, enabling the evaluation of antimicrobial photobiological activity.

**Figure 6:**
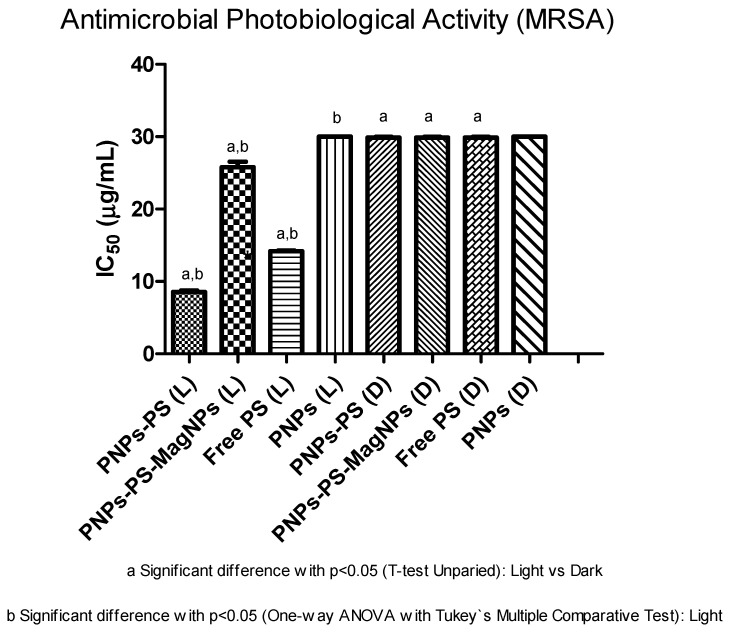
Comparative evaluation of the antimicrobial photodynamic activity of the nanoparticles against methicillin-resistant *Staphylococcus aureus* (MRSA) under the following conditions: irradiated and non-irradiated; (L) = sample irradiated with light and (D) = without irradiation. Each value represents the mean ± S.D. of six independent determinations (n = 6). IC_50_ values (µg/mL) obtained for PNPs, PNPs-PS, and PNPs-PS-MagNPs are presented in two groups: irradiated (λ = 660 nm; 30 J/cm^2^) and non-irradiated. PNPs-PS, polymeric nanoparticles containing the PS; PNPs-PS-MagNPs, polymeric nanoparticles containing PS and MagNPs; Free PS, Al-Pc-Cl; PNPs, PCL polymeric nanoparticles (drug-free); “a” indicates a statistically significant difference (*p* < 0.05) between irradiated and non-irradiated samples (unpaired *t*-test); “b” indicates a statistically significant difference (*p* < 0.05) among irradiated samples (one-way ANOVA followed by Tukey’s multiple comparisons test).

Antibacterial photobiological activities against MRSA are shown in Table 5 and Figure 6. Promising antibacterial effects were observed, as irradiated PS-PNPs showed an IC_50_ of 8.26 µg/mL, a value lower than the non-irradiated condition, which exhibited an IC_50_ of 30 µg/mL. The antibacterial photobiological activity of irradiated PS-PNPs exhibits a 3.6-fold reduction in IC_50_ (8.26 µg/mL) compared to the non-irradiated condition (IC_50_ = 30 µg/mL) with a statistically significant difference (*p* < 0.05). For PNPs-PS-MagNPs, the IC_50_ reduction was more modest, decreasing from 30 to 26.6 µg/mL under non-irradiated and irradiated conditions, respectively. Free PS showed moderate improvement, with IC_50_ decreasing from 30 to 14.22 µg/mL upon irradiation (*p* < 0.05). PNPs without PS did not exhibit biological activity under either condition. The superior antibacterial photodynamic activity of PNPs-PS compared with PNPs-PS-MagNPs may be associated with modifications induced by the magnetic iron oxide material in the microenvironment, affecting light absorption or PS release. These findings justify further investigations, including increasing PS loading and light dose.

Vancomycin, a reference antibiotic for MRSA infections, demonstrated strong antibacterial activity (IC_50_ = 3.52 µg/mL), a lower value than the irradiated PNPs-PS condition, which exhibited an IC_50_ value of 8.26 µg/mL. The antibacterial action of vancomycin exhibits a 2.3-fold reduction in IC_50_ (3.52 µg/mL) compared to PNPs-PS (IC_50_ = 8.26 µg/mL) with a statistically significant difference (*p* < 0.05). The reference antibiotic was approximately twice as active as PNPs-PS, with a statistically significant difference (*p* < 0.05). Vancomycin acts through inhibition of bacterial cell wall synthesis, a mechanism distinct from Al-Pc-Cl; however, it presents several adverse effects when administered orally and is therefore restricted to hospital use [26]. Photodynamic therapy (PDT) with Al-Pc-Cl may be used in combination with vancomycin for the treatment of topical infections (skin and mucosa) caused by MRSA or other bacteria.

**Figure 7:**
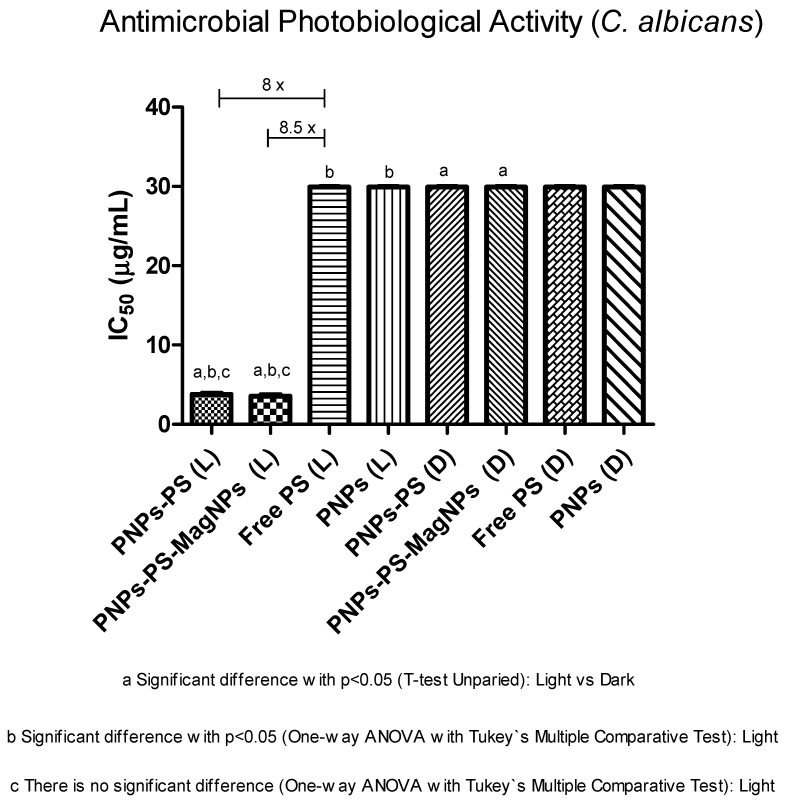
Comparative evaluation of the antimicrobial photodynamic activity of the nanoparticles against *Candida albicans* under the following conditions: irradiated and non-irradiated; (L) = sample irradiated with light and (D) = without irradiation. Each value represents the mean ± S.D. of six independent determinations (n = 6). IC_50_ values (µg/mL) obtained for PNPs, PNPs-PS, and PNPs-PS-MagNPs are presented in two groups: irradiated (λ = 660 nm; 30 J/cm^2^) and non-irradiated. PNPs-PS, polymeric nanoparticles containing the PS; PNPs-PS-MagNPs, polymeric nanoparticles containing PS and MagNPs; Free PS, Al-Pc-Cl; PNPs, PCL polymeric nanoparticles (drug-free); “a” indicates a statistically significant difference (*p* < 0.05) between irradiated and non-irradiated samples (unpaired *t*-test); “b” indicates a statistically significant difference (*p* < 0.05) among irradiated samples (one-way ANOVA followed by Tukey’s multiple comparisons test); “c” indicates that there is no significant difference between irradiated samples (one-way ANOVA followed by Tukey’s multiple comparisons test).

In the evaluation against *Candida albicans*, PNPs-PS and PNPs-PS-MagNPs exhibited strong antifungal activity under irradiation, with IC_50_ values of 3.75 and 3.50 µg/mL, respectively, showing statistically significant differences compared with non-irradiated controls (*p* < 0.05). All non-irradiated samples showed IC_50_ values higher than 30 µg/mL, indicating the absence of significant antifungal effect (Table 5 and Figure 7). Irradiated PNPs-PS (IC_50_ = 3.75 µg/mL) and PNPs-PS-MagNPs (IC_50_ = 3.5 µg/mL) displayed IC_50_ values approximately 8 and 8.8-fold lower than free PS (IC_50_ > 30 µg/mL) (Figure 7). Free PS suffers from instability in aqueous media, undergoing dimerization and loss of photophysical and photobiological properties [8,15].

Fluconazole (reference antifungal) showed an IC_50_ of 8.82 µg/mL against *C. albicans*. The IC_50_ of irradiated PNPs-PS (IC_50_ = 3.75 µg/mL) and PNPs-PS-MagNPs (IC_50_ = 3.5 µg/mL) were approximately twice as low as fluconazole (IC_50_ = 8.82 µg/mL), with a statistically significant difference (*p* < 0.05) (Table 5), representing a significant advance in therapeutic efficacy, particularly in the context of increasing resistance to azole antifungals. These results indicate that encapsulation of the PS into nanoparticles promoted strong antifungal photobiological activity against *C. albicans*, whereas free PS showed no activity. PDT is advantageous because it generates singlet oxygen and other reactive oxygen species (ROS) capable of inactivating microorganisms without inducing antimicrobial resistance mechanisms.

PS-containing nanoparticles have emerged as promising nanocarriers for cancer therapy, particularly in photodynamic therapy (PDT). In this context, the polymeric nanoparticles developed by our research group, PNPs-PS and PNPs-PS-MagNPs, demonstrated significant antitumor photobiological activity against A549 lung cancer cells, reducing cell viability by 42% and 34%, respectively, upon light irradiation. These results highlight the potential of PS-loaded nanosystems to enhance PDT efficacy through improved photosensitizer delivery and cellular interaction. Consistently, several research groups have explored similar nanocarrier strategies to improve the therapeutic performance of photosensitizers in antitumor PDT. For instance, Lumch et al. [27] encapsulated ZnPc in PNPs based in PEGylated Pluronic P123 and poly(L-lactic acid) copolymers. Photobiological studies exhibited efficient internalization of the nanocarrier into tumor cells and superior photodynamic activity compared with free ZnPc. Similarly, Souto et al. [28] reported the incorporation of indium(III) phthalocyanine into nanoparticles. PS-loaded nanoparticles reduced the viability of breast cancer cells by twofold compared with the free photosensitizer at the same dose [29]. In another study, Toledo et al. [29] developed PLGA nanoparticles coated with polyelectrolyte layers to enhance the stability and cellular uptake of zinc(II)-phthalocyanine tetrasulfonate for photodynamic therapy. The nanosystem showed good biocompatibility and significantly improved photodynamic efficacy in B-16 melanoma cells, achieving about 90% tumor cell death after irradiation compared with only 20% for the free photosensitizer. Similarly, Yu et al. [30] developed polymeric nanoparticles capable of efficiently encapsulating ZnPc through aromatic interactions with nicotinate groups. The resulting micelles showed enhanced cellular uptake and significantly increased ROS generation under red-light irradiation, leading to mitochondrial damage and apoptosis in osteosarcoma cells. The ZnPc-loaded micelles exhibited approximately 100-fold higher cytotoxicity than free ZnPc, and in vivo studies demonstrated strong tumor growth inhibition after PDT, highlighting their potential for osteosarcoma therapy. [30].

Nanocarrier systems enhance the solubility, stability, and bioavailability of phthalocyanines while promoting improved interaction with microbial cells and facilitating controlled delivery of the photosensitizer to infection sites. Several studies have demonstrated that phthalocyanine-loaded nanoparticles significantly enhance photodynamic inactivation of bacteria such as *Staphylococcus aureus* and *Escherichia coli*, as well as pathogenic fungi including *Candida albicans*, compared with free PS. Therefore, nanoparticle-mediated delivery of phthalocyanines represents a promising approach for improving the efficacy of antimicrobial photodynamic therapy against drug-resistant bacterial and fungal infections [10,31,32].

Our study presented some limitations, such as weak antibacterial photobiological activity of PNPs-PS-MagNPs (IC_50_ = 25.64 μg/mL) against MRSA. However, in future studies, we will evaluate the antibacterial photobiological activity of the nanocarriers against other species of bacteria. Another limitation was the IC_50_ of irradiated PNPs-PS (IC_50_ = 8.26 μg/mL), which was twice that of vancomycin (the reference antibiotic) (IC_50_ = 3.51 μg/mL). However, we must understand that the drugs have different mechanisms of action, but treatments can be combined with oral administration of the antibiotic and topical PDT for cases of severe infections.

Nanocarriers containing the photosensitizer aluminum phthalocyanine chloride (Al-Pc-Cl) exhibit significant photobiological activity, including antitumor, antibacterial, and antifungal effects. Therefore, future studies will investigate the therapeutic potential of these nanocarriers under light irradiation in additional tumor models, including skin cancers (melanoma and non-melanoma), as well as prostate, breast, head, and neck cancers. Furthermore, the photobiological activity of the PS-loaded nanocarriers will be evaluated against pathogenic microorganisms of clinical relevance, particularly *Leishmania* spp. and *Sporothrix* spp., in order to explore their potential application in the photodynamic treatment of parasitic and fungal infections.

## 4. Conclusions

MagNPs were successfully produced and stabilized with an oleic acid coating. Both MagNPs and PNPs-PS-MagNPs were attracted by a magnetic field, confirming their magnetic properties. Nanoparticles were successfully obtained using the emulsion–solvent evaporation technique, which proved suitable for efficient encapsulation of both the PS and MagNPs. The nanoparticles presented sizes within the nanometric range, with PdI values compatible with monodisperse systems, indicating that the method was appropriate for producing PNPs-PS and PNPs-PS-MagNPs. Process yield, encapsulation efficiency, and PS loading in the nanocarriers were also satisfactory. The PNPs promoted slow and sustained PS release with kinetics consistent with the Higuchi model, indicating the presence of a diffusion-controlled release mechanism.

The nanoformulations exhibited both antitumoral and antimicrobial photobiological activities. PNPs-PS and PNPs-PS-MagNPs were not cytotoxic, presenting cell viabilities above 70% in the MTT assay. Due to this low cytotoxicity, antitumoral photobiological studies using A549 (human lung adenocarcinoma) cells were successfully performed. Upon irradiation, PNPs-PS and PNPs-PS-MagNPs showed strong antitumoral activity, reducing cell viability to approximately 42% and 34%, respectively. PNPs-PS-MagNPs offer the additional advantage of magnetic targeting to a specific tissue using an external magnetic field.

The antibacterial photobiological activity of PNPs-PS was excellent and superior to that of PNPs-PS-MagNPs and free PS. Antibacterial photobiological activity of PNPs-PS was successfully demonstrated against MRSA, with promising IC_50_ values comparable to the reference antibiotic vancomycin. Furthermore, PNPs-PS-MagNPs and free PS showed weak and modest antibacterial photobiological activity against MRSA, respectively. The authors will conduct future studies with nanocarriers using other bacteria, such as Enterococci and Pseudomonas.

The antifungal photobiological activities of PNPs-PS and PNPs-PS-MagNPs were excellent and superior to the activity of free PS. Furthermore, PNPs-PS and PNPs-PS-MagNPs exhibited antifungal photobiological activity against *C. albicans*, with IC_50_ values lower than the reference antifungal fluconazole. The promising results observed for antifungal photobiological activity provide a strong rationale for further investigations against clinically relevant and difficult-to-treat fungal pathogens, such as *Candida auris* and *Sporothrix* spp. These emerging and neglected pathogens are associated with significant therapeutic challenges due to their intrinsic or acquired resistance to conventional antifungal agents, highlighting the potential of this approach as a promising alternative strategy for antifungal therapy.

Topical PDT may therefore be used in combination with chemotherapy for cancer treatment, antibiotics for bacterial infections, and antifungals for resistant fungal infections, representing a versatile and synergistic therapeutic strategy.

## Figures and Tables

**Table 1:** Size, PdI and Zeta potential.

Sample	Size (nm)	PdI	Zeta Potential (mV)
MagNPs	33.6 ± 9.6	0.110 ± 0.012	−12.42 ± 0.51
PNPs	186.3 ± 2.951	0.073 ± 0.008	−2.98 ± 0.12
PNPs-PS	186.9 ± 1.153	0.050 ± 0.023	−4.75 ± 0.32
PNPs-PS-MagNPs	333.56 ± 2.145	0.078 ± 0.028	−5.27 ± 026

MagNPs, magnetic nanoparticles; PNPs, PCL polymeric nanoparticles; PNPs-PS, polymeric nanoparticles containing photosensitizer; PNPs-PS-MagNPs, polymeric nanoparticles containing photosensitizer and magnetic nanoparticles; PNPs, polymeric nanoparticles; PdI, polydispersity index; PdI < 0.3 indicates a monodisperse system with a narrow, low size distribution. Results are expressed as the mean ± S.D. of n = 3 determinations.

**Table 2:** Process yield (Y), encapsulation efficiency (EE), and drug content (DC).

Sample	Y (%)	EE (%)	DC (µg Al-Pc-Cl/mg PNPs)
PNPs-PS	75 ± 1.45	95 ± 3.71	2.92 ± 0.25
PNPs-PS-MagNPs	91 ± 0.78	87 ± 2.23	2.35 ± 0.37

DC, drug content; MagNPs, magnetic nanoparticles; PNPs, PCL polymeric nanoparticles; PNPs-PS, polymeric nanoparticles containing photosensitizer; PNPs-PS-MagNPs: polymeric nanoparticles containing photosensitizer and magnetic nanoparticles. Results are expressed as the mean ± S.D. of n = 3 determinations.

**Table 3:** Correlation coefficient (r) obtained after applying the mathematical models to the in vitro release data.

Sample	Zero-Order	Higuchi	First-Order
PNPs-PS	0.8967	0.9914	0.8178
PNPs-PS-MagNPs	0.8382	0.9901	0.8009

PNPs-PS: polymeric nanoparticles containing photosensitizer; PNPs-PS-MagNPs: polymeric nanoparticles containing photosensitizer and magnetic nanoparticles.

**Table 4:** Cell viability (%) and antitumor photobiological activity of nanoparticles in the A549 cell line under irradiation and not irradiated.

Samples	Cellular Viability (%) (Irradiated)	Cellular Viability (%) (Not Irradiated)
PNPs-PS	42.05 ± 2.17 ^a,b^	82.98 ± 3.11 ^a^
PNPs-PS-MagNPs	34.11 ± 1.75 ^a,b,c^	75.51 ± 2.85 ^a^
Saline	98.72 ± 2.21 ^b,c^	92.88 ± 2.72
PNPs	97.12 ± 0.87 ^b,c^	98.72 ± 2.21

Each value represents the mean ± S.D. of six independent determinations (n = 6). Note: “a” indicates a statistically significant difference (*p* < 0.05) between irradiated and non-irradiated samples (unpaired *t*-test); “b” and “c” indicate a statistically significant difference (*p* < 0.05) between irradiated samples (one-way ANOVA followed by Tukey’s multiple comparisons test).

**Table 5:** Results of antimicrobial photobiological activity expressed as IC_50_ values for different strains of microorganisms irradiated with light and non-irradiate.

Microorganisms	Samples	IC_50_ (μg/mL) (Irradiated)	IC_50_ (μg/mL) (Not Irradiated)
MRSA	PNPs-PS	8.26 ± 0.45 ^a,b,c^	>30 ^a^
	PNPs-PS-MagNPs	25.64 ± 7.12 ^a,b,c^	>30 ^a^
	Free PS	14.22 ± 1.15 ^a,b,c^	>30 ^a^
	PNPs	>30	>30
	Vancomicina	-	3.51 ± 0.31 ^c^
*Candida albicans*	PNPs-PS	3.75 ± 0.4 ^a,b,c,d^	>30 ^a^
	PNPs-PS-MagNPs	3.5 ± 0.03 ^a,b,c,d^	>30 ^a^
	Free PS	>30 ^b^	>30
	PNPs	>30	>30
	Fluconazol	-	8.82 ± 0.26 ^c^

Dosimetry: the irradiated group received a dose of red light at a wavelength of 660 nm, with an irradiance of 30 J/cm^2^, using the DMC^®^ Photon Lase I device. Results were expressed as mean ± SD from n = 6 determinations. MRSA, methicillin-resistant *Staphylococcus aureus*; PNPs-PS, polymeric nanoparticles containing the photosensitizer; PNPs-PS-MagNPs, polymeric nanoparticles containing the photosensitizer and magnetic nanoparticles; PNPs, PCL-based polymeric nanoparticles (blank); Free PS, Al-Pc-Cl solution; “a” indicates statistically significant difference (*p* < 0.05) between non-irradiated and irradiated samples (unpaired *t*-test); “b” indicates statistically significant difference (*p* < 0.05) among irradiated samples (one-way ANOVA with Tukey’s multiple comparison test); “c” indicates statistically significant difference (*p* < 0.05) between irradiated nanoparticle samples containing PS and vancomycin (reference antibiotic) or fluconazole (reference antifungal) (one-way ANOVA with Tukey’s multiple comparison test); “d” indicates that there is no statistically significant difference between irradiated samples (unpaired *t*-test).

## Data Availability

The original contributions presented in this study are included in the article/Supplementary Materials. Further inquiries can be directed to the corresponding author.

## Supplementary Materials

The following supporting information can be downloaded at: https://www.mdpi.com/article/10.3390/cimb48030324/s1.

## Abbreviations

The following abbreviations are used in this manuscript:

DCM	dichloromethane
PVA	polyvinyl alcohol
PMMA	poly-(methyl methacrylate)
PDT	photodynamic therapy
PS	photosensitizer
PC	phthalocyanine
Al-Pc-Cl	aluminum phthalocyanine chloride
NPs	nanoparticles
ZnPc	zinc phthalocyanine
PNPs	polymeric nanoparticles
PCL	poly-caprolactone
MagNPs	magnetic nanoparticles
TEM	transmission electronic microscopy
PdI	polydispersity index
