# Supplementary material for: Evaluation of Antitumor and Antimicrobial Photobiological Activity of Nanocarrier Containing Photosensitizer and Magnetic Nanoparticle
Source: Curr Issues Mol Biol. 2026 Mar 19;48(3):324. doi: 10.3390/cimb48030324 (PMC13024851; doi:10.3390/cimb48030324)
Supplement: Supplementary file 1 [file cimb-48-00324-s001.zip › cimb-4198288-supplementary.pdf]

Supplementary Materials

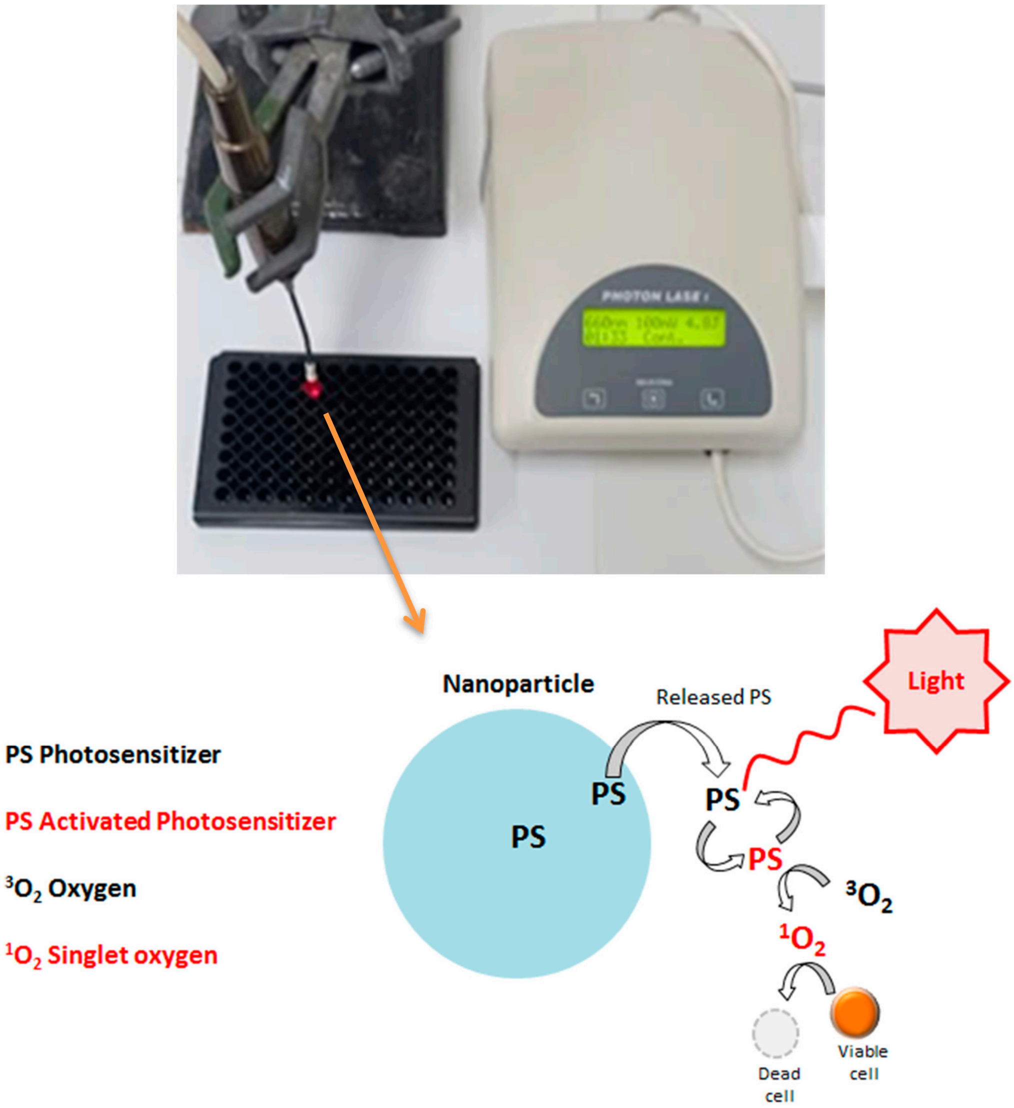

Figure S1. Light source for evaluating photobiological activity.

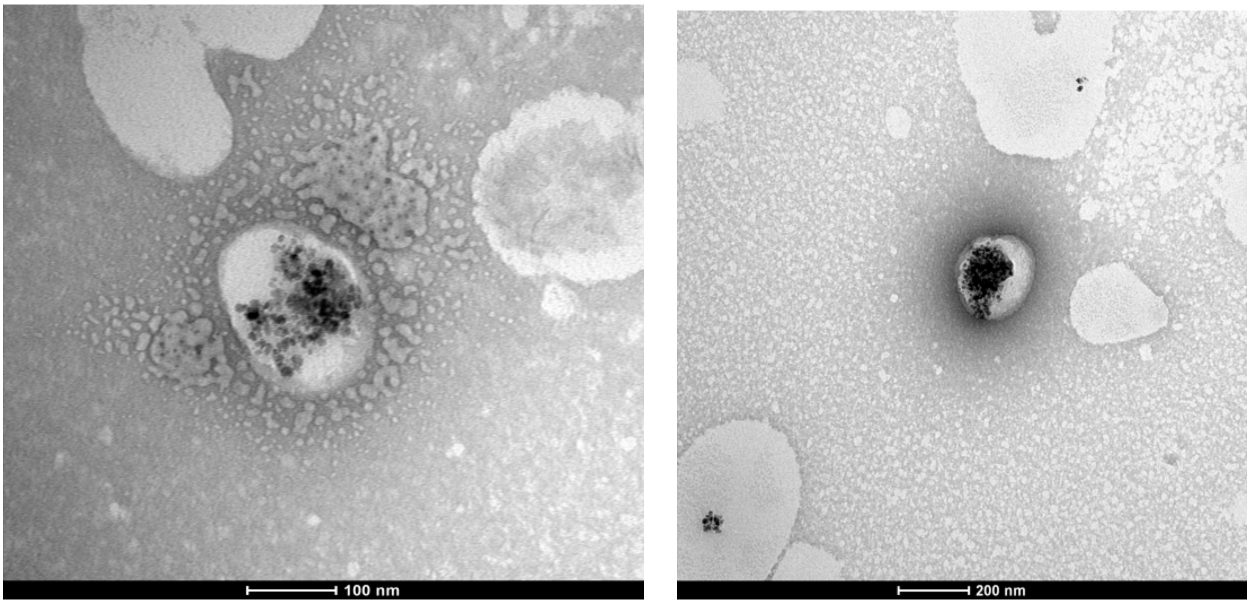

Figure S2. TEM images of PNPs-PS-MagNPs: black dots indicate MagNPs loaded on polymeric nanoparticles.

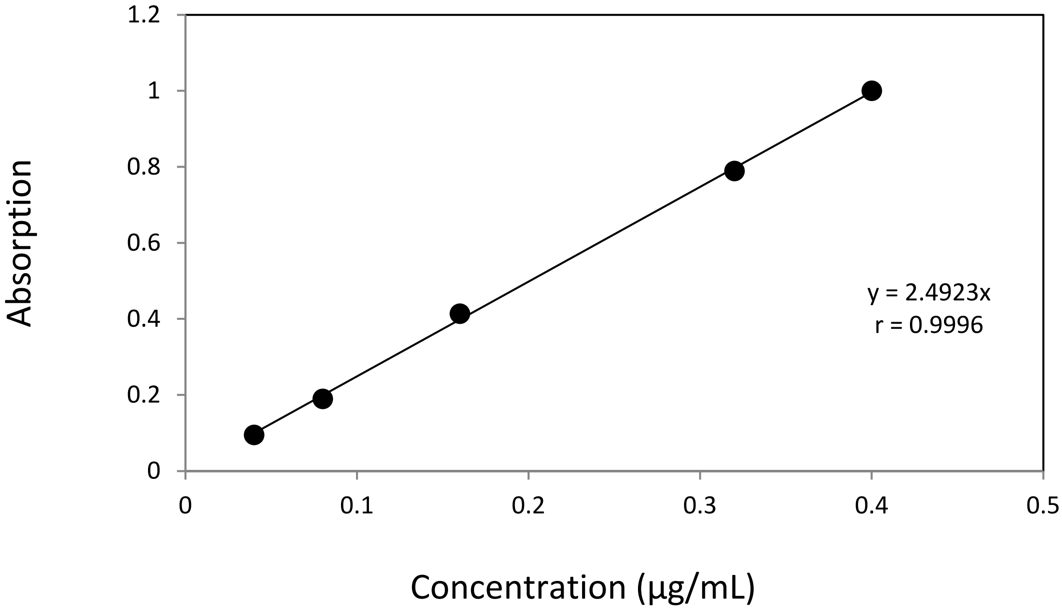

Figure S3. Analytical curve of PS in ethanol.

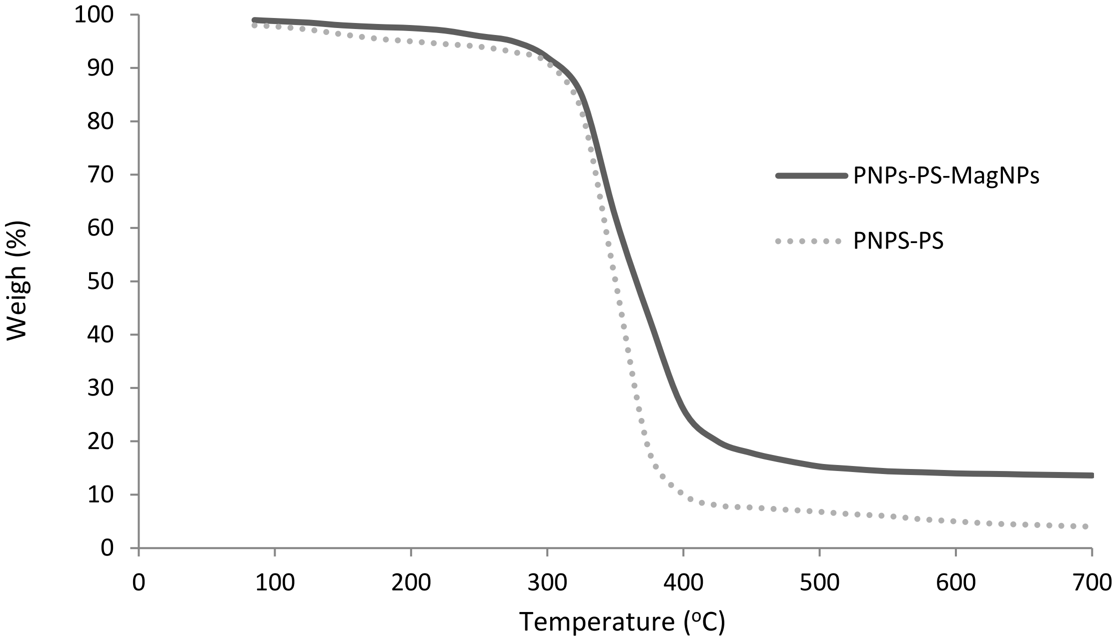

Figure S4. Thermogravimetric analysis obtained for PNPs-PS and PNPs-PS-MagNPs.
